# Aquatic polymers can drive pathogen transmission in coastal ecosystems

**DOI:** 10.1098/rspb.2014.1287

**Published:** 2014-11-22

**Authors:** Karen Shapiro, Colin Krusor, Fernanda F. M. Mazzillo, Patricia A. Conrad, John L. Largier, Jonna A. K. Mazet, Mary W. Silver

**Affiliations:** 1 One Health Institute, School of Veterinary Medicine, University of California, Davis, CA 95616, USA; 2 Department of Pathology, Microbiology and Immunology, School of Veterinary Medicine, University of California, Davis, CA 95616, USA; 3 Bodega Marine Laboratory and Department of Environmental Science and Policy, University of California, Davis, CA 95616, USA; 4 Department of Ocean Sciences, University of California, Santa Cruz, CA 95064, USA

**Keywords:** extracellular polymeric substances, transparent exopolymer particles, *Toxoplasma gondii*, zoonotic pathogen, marine transmission, sea otter

## Abstract

Gelatinous polymers including extracellular polymeric substances (EPSs) are fundamental to biophysical processes in aquatic habitats, including mediating aggregation processes and functioning as the matrix of biofilms. Yet insight into the impact of these sticky molecules on the environmental transmission of pathogens in the ocean is limited. We used the zoonotic parasite *Toxoplasma gondii* as a model to evaluate polymer-mediated mechanisms that promote transmission of terrestrially derived pathogens to marine fauna and humans. We show that transparent exopolymer particles, a particulate form of EPS, enhance *T. gondii* association with marine aggregates, material consumed by organisms otherwise unable to access micrometre-sized particles. Adhesion to EPS biofilms on macroalgae also captures *T. gondii* from the water, enabling uptake of pathogens by invertebrates that feed on kelp surfaces. We demonstrate the acquisition, concentration and retention of *T. gondii* by kelp-grazing snails, which can transmit *T. gondii* to threatened California sea otters. Results highlight novel mechanisms whereby aquatic polymers facilitate incorporation of pathogens into food webs via association with particle aggregates and biofilms. Identifying the critical role of invisible polymers in transmission of pathogens in the ocean represents a fundamental advance in understanding and mitigating the health impacts of coastal habitat pollution with contaminated runoff.

## 1. Introduction

While contamination of near-shore waters with pathogens from terrestrial hosts is a threat to human and animal health, the mechanisms of disease transmission in marine ecosystems are rarely addressed [1]. Specifically, investigations addressing the role of excreted polymers in transmission of terrestrially derived (henceforth termed terrigenous) pathogens are lacking, despite numerous studies documenting their critical functions in aquatic habitats. Polymers such as extracellular polymeric substances (EPSs) represent a continuum of dissolved to particulate, gelatinous, sticky organic molecules [2] that are essential for many life processes in fresh, estuarine and marine waters, including biogeochemical cycling and feeding, locomotion and defence of organisms [3]. Here, we integrate findings from the disciplines of oceanography, ecology and epidemiology into those from parasitology in order to investigate the role of polymers in transmission of a model terrestrial pathogen, *Toxoplasma gondii*, in marine environments. *Toxoplasma gondii* is a ubiquitous protozoan parasite that infects animals and humans (i.e. zoonotic pathogen) worldwide [4]. Although intermediate hosts for *T. gondii* include numerous warm-blooded animals, only felids serve as definitive hosts, with sexual reproduction of the parasite resulting in formation of millions of oocysts shed in faeces [5]. *Toxoplasma gondii* oocysts can persist in the environment for months to years [6] and are sources of infection for marine mammals, including the threatened southern sea otter (*Enhydra lutris nereis*) in California [7,8]. Infections with *T. gondii* in humans are acquired by several routes, including ingestion of contaminated water or undercooked shellfish, and may be asymptomatic or lead to disseminated disease and death [9].

Given the considerable dilution of runoff and pathogens upon delivery to coastal waters, the high *T. gondii* prevalence in sea otters and widespread infections in diverse marine mammal species are puzzling and raise the question of how terrigenous pathogens are so effectively transmitted to marine fauna. The curious association of high *T. gondii* exposure in sea otters having a dietary preference for marine snails [10] suggests that specific transport mechanisms of the parasite in near-shore waters may be driving transmission of this potentially lethal parasite to higher-trophic-level predators. In fact, otters that specialize on predation of kelp-dwelling snails have more than 10-fold higher odds for infection with *T. gondii* when compared with otters that share the exact same spatial location but prefer other prey [10]. However, to date, the fate of *T. gondii* oocysts that are deposited in marine waters through contaminated freshwater runoff has not been investigated, and the mechanisms that render snails more likely to transmit this terrigenous parasite to otters compared with other invertebrate prey are unknown.

Sticky polymeric substances could significantly impact the aquatic transport of pathogens such as *T. gondii* and their subsequent incorporation into food webs via two distinct processes: enhanced association with drifting marine macroaggregates (e.g. marine snow) [11] and direct adhesion to biofilms on benthic structures, including seaweeds [3]. Pelagic aggregates are ubiquitous particles in natural waters, derived from the spontaneous assembly of dissolved and particulate materials, as well as from mucous products of a wide variety of prokaryotic and eukaryotic organisms [12]. While decades of research demonstrate that macroaggregates are involved in important biological and physical processes in aquatic ecosystems [11,13–15], studies investigating their role in disease transmission are limited. Aggregates have been found to concentrate non-zoonotic pathogens, faecal indicator bacteria and opportunistic bacteria [16,17]. Although the association of zoonotic pathogens, including *T. gondii,* with macroaggregates has been demonstrated under laboratory conditions, observations in natural environments are difficult to acquire and environmental factors conducive to this association are poorly known [18,19]. Because macroaggregates (marine snow) tend to settle faster than their individual small component particles and serve as food for many organisms, the association of pathogens with macroaggregates profoundly impacts disease transmission dynamics in two ways: (i) by altering the spatial distribution of microscopic pathogens, and thus the spatial patterns of host exposure to these disease agents; and (ii) by increasing the effective particle size, and thus enhancing bioavailability to invertebrate consumers [20], which in turn can vector these agents to predators at higher trophic levels, including humans.

**Figure 1.**
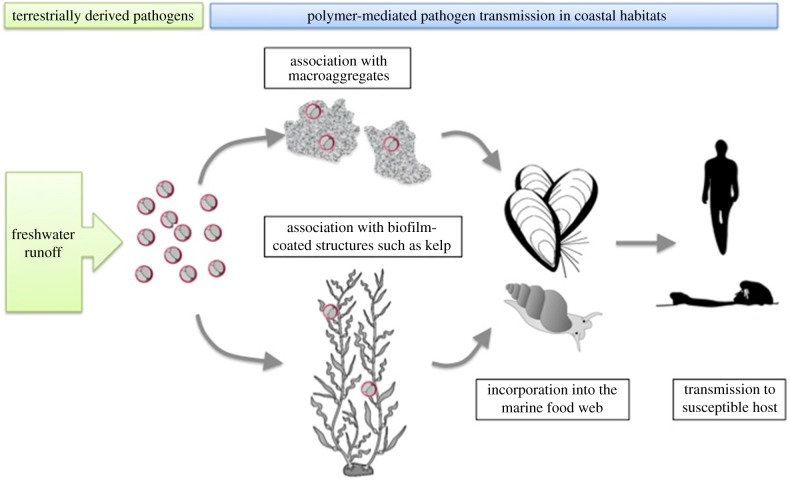
Two mechanisms are proposed whereby polymers can mediate transmission of terrestrial pathogens in coastal ecosystems (depicted here for a model zoonotic protozoan parasite, *Toxoplasma gondii*). Pelagic polymers such as TEPs may enhance the association of pathogens with sinking macroaggregates (shown in this study), while benthic exopolymer substances (EPS) can trap pathogens within sticky biofilms [22]. Both mechanisms are likely to increase the probability of pathogen entry into the marine food web through aggregate-consuming invertebrates such as bivalves or surface scraping molluscs such as snails. Ingestion of contaminated prey items can then lead to pathogen exposure in susceptible hosts, including threatened California sea otters and humans.

In this study, we investigate the effect of transparent exopolymer particles (TEPs), a particulate form of EPS that serves as ‘glue’ in aggregation processes [21], on the likelihood of *T. gondii* association with marine aggregates (i.e. a pelagic aggregation mechanism). Recent work has highlighted the chemical and physical role of TEPs and other gelling agents in coagulating materials, with the resulting particles ranging from one to hundreds of micrometres, thereby promoting the assembly of even larger, marine snow-sized aggregates [14,15]. Results from aggregation studies were then combined with recent findings that demonstrate the role of EPS in attaching *T. gondii* to benthic macroalgal surfaces [22], which are subsequently grazed and retained by kelp-dwelling turban snails [23] that consume and thereby vector the parasite to sea otter predators (i.e. a benthic biofilm mechanism). Together, these two polymer-mediated mechanisms represent a significant and previously unrecognized bio-concentration process that both alters the spatial distribution of disease agents in marine ecosystems and increases the likelihood of susceptible host exposure to terrigenous disease agents (figure 1). The case study of *T. gondii* transmission described here provides compelling evidence for the fundamental but previously unrecognized role of polymer-mediated disease transmission in coastal waters.

## 2. Material and methods

### (a) Aggregation experiments

#### (i) Water samples

Two separate experiments were conducted to test the aggregation of suspended *T. gondii* oocysts and surrogate microspheres in seawater samples as a function of a single type of TEP concentration (experiment 1), as well as in natural seawater collected under differing dominant phytoplankton conditions that would presumably exude TEPs of differing molecular composition and size (experiment 2). For experiment 1, a single surface seawater sample (20 l) was collected from Santa Cruz pier (36.957° N, 122.017° W) using a bucket. The same site was used to collect three water samples for experiment 2 in spring and summer, when different diatom communities were present (table 1). Experiment 1 was conducted to evaluate the specific effect of a single type of TEP on particle aggregation by using seawater spiked with increasing concentrations of alginic acid (produced by *Macrocystis pyrifera* and provided commercially by Sigma-Aldrich, St Louis, MO, USA) to simulate TEP concentrations at levels equivalent to those observed under natural conditions [15]. This approach yielded water samples that were identical in their physico-chemical properties except for the controlled difference in TEP produced by the addition of alginic acid. The physico-chemical properties of the different water types used in experiments 1 and 2 are listed in table 1.

**Table 1.** Physico-chemical properties, phytoplankton abundance and TEP concentrations in water samples used in aggregation experiments 1 (March) and 2 (March, June and August). chl *a*, chlorophyll *a*; ppt, parts per thousand; NTU, nephelometric turbidity units; TSS, total suspended solids; TSS-C, TSS carbon component; TSS-N, TSS nitrogen component; Xeq, xanthan gum equivalent.

month (2012)	dominant phytoplankton	cell count ml^−1^	chl *a* µg l^−1^	salinity (ppt)	pH	turbidity (NTU)	TSS (mg l^−1^)	TSS-C (mg l^−1^)	TSS-N (mg l^−1^)	TEP (µg Xeq. l^−1^)
March	*Skeletonema* cf. *costatum*	300	n.a.	32.2	8.0	13	3.4	0.5	<0.1	332
June	*Chaetoceros* spp.	15 000	14.3	29.0	8.3	4	5.3	1.05	0.14	373
August	*Pseudonitzschia* spp*. seriata* group	139	4.5	33.1	8.1	2	6.2	0.28	0.03	164

#### (ii) Transparent exopolymer particle and chlorophyll *a* quantification, and phytoplankton species composition

A subsample of the surface seawater collected at Santa Cruz Pier (above) for aggregation experiments via a bucket was aliquoted for quantification of TEPs and chlorophyll *a*, and for enumeration of dominant phytoplankton taxa*.* Samples for chlorophyll *a* were immediately filtered onto glass fibre filters (GF/F 0.7 pore size 47 mm diameter). Two replicates of 25 ml were filtered onto GF/F filters and kept in a −20°C freezer. Chlorophyll *a* was extracted for 24 h in 90% acetone and subsequently analysed on a Turner Design 10AU fluorometer. For TEP quantification, aliquots of 1 l were preserved with formaldehyde to achieve a 1% final concentration, and then stored at 4°C in the dark for later analysis. TEPs were measured by filtering up to three replicates of 100–250 ml aliquots onto 0.4 µm polycarbonate filters. Concentrations were determined using a standard semi-quantitative colorimetric assay and a UV-1201 UV-VIS spectrophotometer set at 787 nm [24]. A calibration curve using xanthan gum as the standard was constructed to generate a conversion factor (*F*-factor) to relate the absorbance of stained TEPs to the weight of TEPs [24].

In addition to collecting surface water using buckets, samples were also obtained from the same location using a net (35 µm mesh) to assess phytoplankton composition. Sampling took place during daylight hours at approximately 20–30 cm below the surface. Live net tow material was examined in the laboratory using an inverted microscope (Olympus IMT-2). The net phytoplankton community (cells ≥ 20 μm) was characterized by microscopy and taxa identified using morphological appearance to genus or species level. The relative contribution of individual taxa to the total net phytoplankton community was then estimated. The ‘dominant taxon’ is defined here as the one contributing more than 45% of the total phytoplankton cells in the net sample. Taxa were identified to genus level to avoid misidentification of species, but tentative species designations were indicated. For enumeration of dominant phytoplankton taxa, aliquots of 100 ml from bucket samples were preserved in 4% formalin final concentration and stored in the dark at 4°C. Enumeration was done in aliquots of 10 ml settled for 24 h in an Utermöhl chamber following the Utermöhl method [25]. A minimum of 100 cells was counted per chamber using an inverted microscope (Olympus IMT-2).

#### (iii) *Toxoplasma gondii* oocysts and surrogate microspheres

*Toxoplasma gondii* (Type II) oocysts were produced using previously described methods [26]. Owing to the biohazardous nature of *T. gondii*, oocysts were heat-inactivated prior to use in aggregation and snail exposure experiments by immersion in 80°C dry bath for 20 min. Aggregation behaviour of two types of autofluorescent microspheres, previously validated as *T. gondii* surrogate particles [27], were also evaluated in this study: Dragon green (10.35 µm) and glacial blue (8.6 µm) carboxyl-modified polystyrene microspheres were obtained from Bangs Laboratories (Fishers, IN; product numbers FC07F 5493 and PC06N 8319, respectively). In the snail exposure experiment, only dragon green microspheres were used as comparison to ingestion and retention of *T. gondii* oocysts.

#### (iv) Aggregate production

A rolling apparatus was used to rotate cylindrical water bottles, to produce aquatic aggregates under laboratory conditions [28]. In each experiment, water samples (450 ml) were placed in glass bottles (filled to the rim to minimize air space), spiked with 4500 *T. gondii* oocysts and 4500 of the surrogate microspheres, and then placed on the rolling apparatus for 24 h. Aggregation control bottles consisting of filtered seawater and corresponding to each water type tested were processed in an identical fashion as non-filtered samples to evaluate particle distribution in the absence of material that could lead to aggregate formation. Five replicates of non-filtered and three replicates of filtered-control bottles were used for each water type.

#### (v) Aggregate analyses and particle quantification

After 24 h, bottles were placed upright for 20 min to allow the readily visible aggregates to settle, and the top 400 ml—operationally defined ‘aggregate-poor’ bulk water [16]—was gently removed. A photograph was obtained through the mouth of the open bottle to image the particles in the aggregate-rich bottom water fraction that remained (50 ml). Quantification of the total number and volume of snow-sized (more than 0.5 mm) aggregates was performed by analysing the images of the settled aggregates in the bottom water fraction. Bottles were placed on a black background with a 10 mm-marked grid for particle size calibration, and a photograph taken using a Canon SD1200 IS digital camera. Photographs were processed as previously described by Lyons *et al*. [29] using ImageJ image analysis software (W. S. Rasband, ImageJ, US National Institutes of Health, http://imagej.nih.gov/ij). The volume of aggregates was estimated using a right cylinder equation derived from short and long axis measurements [28]. *Toxoplasma gondii* oocysts and surrogate microspheres were quantified in aggregate-poor and aggregate-rich water fractions separately, using a membrane filtration technique as previously described [30].

#### (vi) Visualization of transparent exopolymer particle-entrained *Toxoplasma gondii*

For evaluating the presence of *T. gondii* oocysts within aggregates and snail faecal pellets, a qualitative method for visualizing matrices containing TEPs was performed by staining an aliquot of aggregate-rich fractions or faecal pellet slurries with alcian blue. Staining was performed as described by Alldredge *et al*. [11] and the slides were microscopically evaluated under a combination of bright field illumination and DAPI excitation using a Zeiss Axioskop epifluorescent microscope equipped with a UV emission filter set (emitter 460, 50 nm band pass filter; Chroma 11000v3).

#### (vii) Aggregation experiment data analyses

To analyse data from the *T. gondii* aggregation experiments (1 and 2), a univariable negative binomial regression model was fitted to assess whether TEP concentration was a significant predictor of the numbers of *T. gondii* oocysts and surrogates recovered from the aggregate-rich fraction of a sample. An aggregate enrichment factor (EF) was also estimated to quantify the magnitude of increased numbers of oocysts or surrogates in the aggregate-rich fraction that was due to adherence to, or incorporation within, aquatic aggregates [12]. Particle EFs were calculated for each bottle using the following equation:



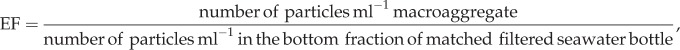


where the numerator was calculated using the following equation:



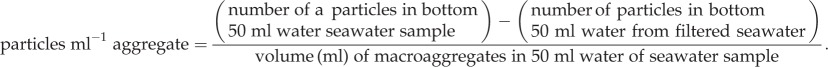


All statistical analyses were performed using STATA (College Station). Significance was indicated when *p* ≤ 0.05 for all statistical analyses. All data generated from the aggregation experiments are publicly available in Dryad data repository [31].

### (b) Polymer-mediated uptake of *Toxoplasma gondii* by marine snails

The methods employed for determining the quantitative association of *T. gondii* surrogates with kelp biofilms are described in detail by Mazzillo *et al*. [22].

#### (i) Snail collection and experimental set-up

To evaluate the potential role of marine snails in vectoring *T. gondii* from contaminated kelp surfaces, a laboratory exposure study was conducted using three species of turban snails (*Chlorostoma brunnea, C. montereyi* and *Promartynia pulligo*) known to serve as prey for sea otters. A cross-sectional sample of 81 brown turban snails, along with kelp blades, was collected by divers from kelp surfaces near Cambria, CA, USA. The snails were shipped overnight to the University of California, Davis laboratory, where they were weighed, rinsed and sorted by species. Four polyethylene tubs were filled with 4 l filtered (0.2 µm) seawater and labelled A, B, C or D. Kelp blades were rinsed, wiped with paper towels and added to each tub. Individuals from each species group were sequentially allocated to tubs A, B and C. Each of these three tubs received a group of 24 snails having the same species distribution as the field sample. The fourth tub (D) was maintained without snails to evaluate oocyst distribution in the tub in the absence of kelp grazers. All four tubs were maintained at 10–12°C for the durations of the acclimation and experimental phases of the study. During the initial 72 h acclimation period, all faecal material was removed from tubs containing snails, and all kelp and half of the water volume were replaced every 24 h. Following acclimation, the kelp blades and half of the seawater volume of each tub was replaced, and tubs A, B and D were spiked with heat-inactivated *T. gondii* oocysts to a final concentration of 10^4^ oocysts per litre. Tub C was not spiked with oocysts and served as a negative control treatment.

#### (ii) Depuration period

Twenty-four hours following spiking of tubs A, B and D, the snails were removed from the tubs, rinsed and transferred to individual 1 l acrylic boxes containing 800 ml filtered seawater. The boxes were furnished with a fresh piece of washed kelp and an air tube. No exchange of water occurred between boxes. All faecal pellets were removed from the tubs using serologic pipettes, and the total volumes of faecal pellets recovered from each tub were recorded. From the snail-free control tub (D), a volume of seawater equal to the mean faecal pellet suspension volume recovered from the snail-containing tubs was removed from the bottom using a serologic pipette. Following introduction of the snails into the individual boxes, kelp pieces were removed from each box using forceps and all faecal material was aspirated using separate serologic pipettes for each box every 24 h for a total depuration period of 14 days. The entire volume of filtered seawater was replaced and fresh kelp added to each box following thorough rinsing of the snail and the box every day.

#### (iii) Quantification of *Toxoplasma gondii* in snail faeces

The suspensions of dark green, rod-like faecal pellets collected from the 4 l tubs following the 24 h exposure period, and from each box through the 14-day follow-up period, were transferred to conical-bottom 50 ml polypropylene tubes, and homogenized using 15-gauge needles. Faecal samples were vortexed and aliquots diluted for membrane filtration and microscopy for oocyst quantification, following methods as described above for aggregation studies [30]. Dilution, filtration and microscopy continued for each sample until at least five *T. gondii* oocysts had been observed or until the entirety of the sample had been examined. The total number of *T. gondii* oocysts in the sample was then estimated as the product of the observed number of oocysts and the inverse of the fraction of the sample that was processed. The estimate of the total numbers of *T. gondii* oocysts in the faecal samples collected from the spiked tubs at the end of the 24 h exposure period was corrected by subtraction of the number of oocysts observed in the bottom water fraction sample from the snail-free control tub (D), which was filtered and examined in its entirety. This correction was necessary in order to exclude any oocysts that had not been ingested and excreted by a snail from the estimates of oocyst numbers passed in faecal pellets from snails in the exposure tubs.

## 3. Results and discussion

### (a) Aggregation mechanism: role of transparent exopolymer particles in associating *Toxoplasma gondii* with marine snow

**Figure 2.**
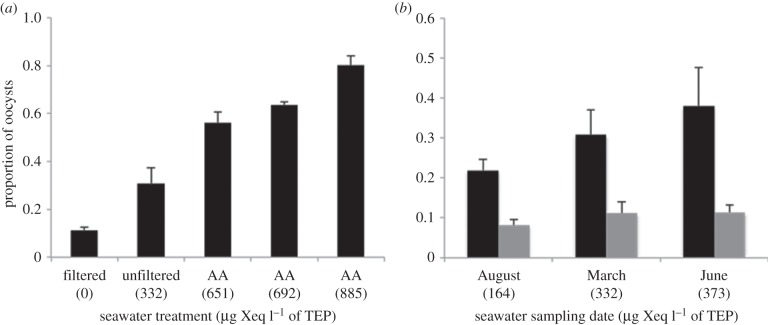
Proportion of *Toxoplasma gondii* oocysts recovered after 24 h from the aggregate-rich fractions of seawater with differing TEP concentrations in (*a*) experiment 1, where seawater was spiked with increasing concentrations of alginic acid (AA; a source of TEP), and (*b*) experiment 2, containing seawater samples collected on different dates with variable but naturally derived TEP mixtures and concentrations. TEP concentrations are indicated in parentheses as μg xanthan gum equivalent l^−1^. In both experiments, significantly higher numbers of oocysts were associated with aggregates as TEP concentrations in the water increased (negative binomial regression *p* < 0.001). Error bars denote 1 s.d. from the mean (*n* = 5). Black bars denote seawater; grey bars, filtered seawater.

To explore the importance of pelagic polymers in mediating pathogen transmission, laboratory aggregation experiments were performed to evaluate whether increasing TEP concentrations enhance the association of *T. gondii* oocysts with marine macroaggregates. In experiment 1, a coastal seawater sample was collected, partitioned into subsamples and spiked with increasing concentrations of alginic acid, an anionic polysaccharide that is produced by a common California macroalgae (*Macrocystis pyrifera*) [32]. Prior research has demonstrated that sticky polysaccharides similar to alginic acid can assimilate into marine gels such as TEPs [33]. Alginic acid was selected as a model polymer in this study because it is commercially available in purified form (extracted from brown algae) and has been previously proposed as a standard for TEP quantification in colorimetric assays [24]. An additional aliquot of seawater was filtered (0.2 μm) to provide a particle-free control. In total, the aggregation of *T. gondii* oocysts and surrogate microspheres (previously validated to have physico-chemical surface properties similar to those of oocysts [27]) were tested in five water types: filtered seawater, seawater containing background levels of TEPs and seawater with three increasing concentrations of TEP added in the form of alginic acid. Results demonstrate increasing *T. gondii* association with aggregates as TEP concentrations in seawater increased, with more than 80% of oocysts associated with aggregates in seawater containing a high concentration of TEP (figure 2*a*). Furthermore, negative binomial regression analysis revealed a significant and positive association between increasing TEP concentrations and the numbers of oocysts associated with aggregates (*p* < 0.001; figure 2*a*).

**Figure 3.**
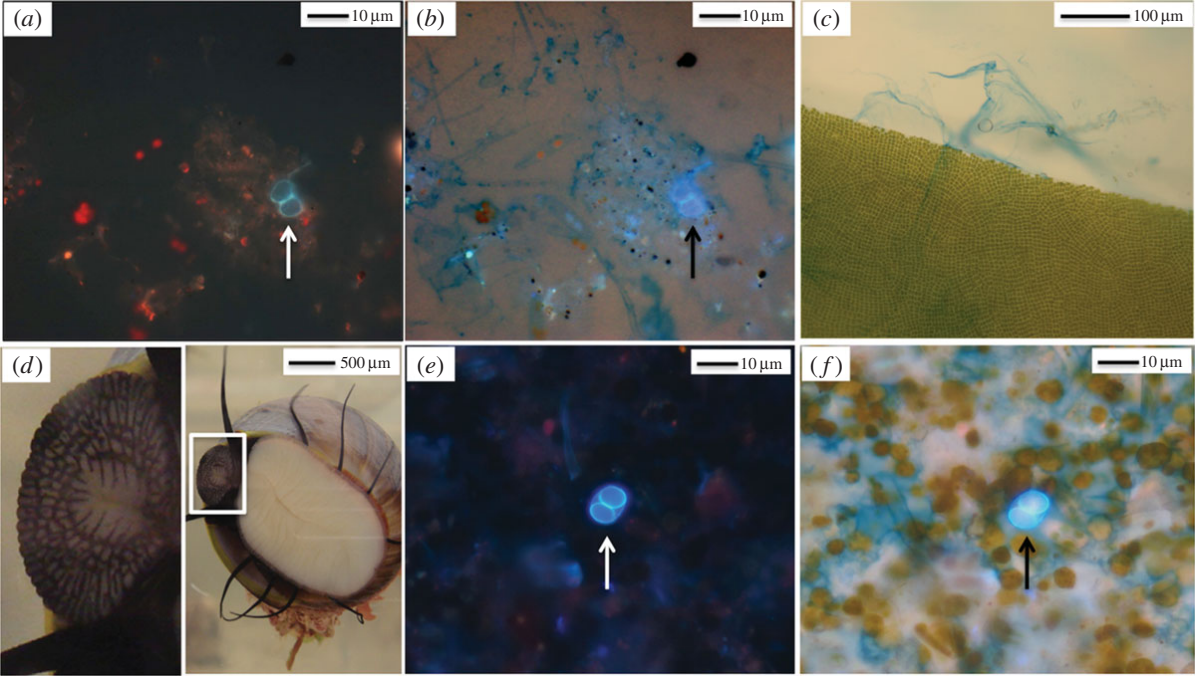
(*a*,*b*) *Toxoplasma gondii* oocyst (arrows) associated with a TEP-embedded aggregate under (*a*) DAPI epifluorescence and (*b*) DAPI and bright field illumination, revealing TEP (blue staining). (*c*) Section of alcian blue-stained kelp showing emanating EPS fibres. (*d*) Ventral surface of turban snail with enlargement of scraping radula. (*e*,*f*) *Toxoplasma gondii* oocyst (arrows) in snail faeces under (*e*) DAPI epifluorescence only and (*f*) combined DAPI and bright field illumination, revealing EPS/TEP (blue) entrained in faecal material.

While the alginic acid experiment demonstrates the role of a single type of TEP in enhancing adhesion of oocysts to aggregates, we also investigated this relationship in seawater containing variable mixtures and concentrations of naturally occurring TEPs. Experiment 2 was conducted by testing *T. gondii* association with aggregates in seawater collected from the Santa Cruz Wharf, CA, USA during times when dominant phytoplankton assemblages differed (table 1). Naturally occurring TEPs were quantified in samples collected in March, June and August 2012 during diatom blooms dominated by *Skeletonema costatum, Chaetoceros* spp. and the *Pseudonitzschia* spp*. seriata* group, respectively. Like the results from experiment 1, our findings indicate that increasing TEP concentrations, including naturally occurring TEPs, are correlated with increasing numbers of oocysts in aggregates (negative binomial regression *p* < 0.001; figure 2*b*). Thus, while the TEP in these three seawater samples probably differed in molecular composition—related to differing phytoplankton communities [34]—a positive relationship was evident between TEP concentrations and *T. gondii* abundance in marine aggregates. Micrographs from these experiments also provided visual evidence of the presence of oocysts in TEP-embedded aggregates (figure 3*a,b*). In both experiments 1 and 2, the degree of association of *T. gondii* oocysts and surrogate microspheres with aggregates was similar in all water samples tested (electronic supplementary material, figure S1 and table S1)—a result that provides a basis for future studies that will use surrogates for investigating the transport and fate of *T. gondii* in natural habitats (a necessary approach, given that the release of biohazardous microorganisms in the field is neither ethically acceptable nor logistically feasible). Quantification of the EF (electronic supplementary material, table S2) highlights the degree to which oocysts and surrogates are associated with marine snow, with particle concentrations three to four orders of magnitude higher in aggregates than in equivalent volumes of water.

As the presence of aquatic polymers such as TEPs varies spatially, their impact on mediating pathogen transmission could yield a spatial pattern of risk for pathogen exposure in coastal waters. High levels of TEPs have been reported in coastal waters receiving freshwater influence, during episodes of phytoplankton blooms or in environments supporting kelp forests [35,36]. Prior investigations suggested that seaweeds produce and release large concentrations of polymeric substances into surrounding waters [3,36]. Using light microscopy, we observed this phenomenon by applying an alcian blue dye that stains EPS fibrils emanating from field-collected kelp (figure 3*c*). Terrigenous pathogens delivered to coastal seawater via freshwater runoff are likely to become associated with marine snow, both due to electrochemical changes occurring as particles move through estuarine habitats [19] and due to the polymer-enhanced aggregation processes demonstrated here. Once associated with aggregates, the settlement rate of pathogens increases, leading to benthic accumulation of pathogens at sites where aggregation processes are enhanced. Following Stokes's law, a 100-fold increase in particle size, such as 10 (single oocyst) to 1000 (macroaggregate) μm, results in a 10 000-fold increase in particle fall velocity. Although the density of a marine aggregate may be less than the density of the particulate pathogen, sinking velocity has a quadratic dependence on size (up to about 1 mm) and a linear dependence on particle–water density difference. Even if a porous macroaggregate has a density equalling 1% of that of the pathogen, the aggregate would still descend 100 times faster. Thus, in shallow waters, an unattached pathogen that would take several days to settle to the sea bed may now do so in a few hours. The aggregation-mediated alteration in the spatial distribution of waterborne pathogens has profound impacts on risk for disease exposure in marine habitats. Benthic filter-feeders, grazers and detritivores are unlikely to become exposed to (and thus unlikely to ingest) individual micrometre-sized pathogens that remain suspended in buoyant, near-surface, freshwater plumes. However, exposure (and hence bioavailability) of terrigenous pathogens to benthic organisms will be greatly enhanced by entrainment of pathogens into larger sinking aggregates. Further, compared with unattached particles, aggregate-associated pathogens (which are now effectively larger in size) are more likely to be ingested and retained by marine invertebrates such as snails and bivalves [20], and thus subsequently transmitted to higher-trophic-level predators, including humans.

### (b) Benthic biofilm mechanism: *Toxoplasma gondii* uptake by kelp-dwelling snails

The second mechanism evaluated recently by our team is one in which mucus-rich polymers facilitate particle adhesion onto kelp surfaces [22], thus enabling pathogen transmission into benthic communities. This finding sheds light on the puzzling pattern of *T. gondii* infections in sea otters: while numerous marine invertebrates that serve as sea otter prey feed on aggregates, only otters that preferentially hunt kelp-dwelling turban snails have increased risk for *T. gondii* infection [10]. Unlike other prey species, these snails graze on kelp surfaces using a scraping radula (figure 3*d*). To test the hypothesis that EPS can facilitate the uptake of *T. gondii* by snails, experiments were performed to evaluate whether *T. gondii* surrogates adhere to kelp surfaces. Employing different tank settings containing either field-collected kelp or model kelp, and either filtered or unfiltered seawater, the association of surrogates with surfaces was assessed in the presence of TEP in the water column, EPS on kelp surfaces or both. Findings revealed that significant numbers of *T. gondii* surrogates could become associated with kelp surfaces when adhesion was promoted by EPS coatings on kelp surfaces [22].

**Figure 4.**
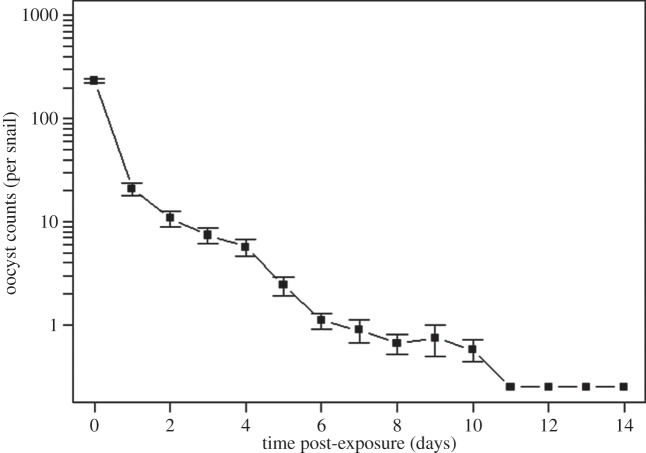
Numbers of *Toxoplasma gondii* oocysts detected in snail faeces over 14 days in which snails were maintained in an oocyst-free environment. Following the 24 h exposure period (time 0), oocysts were present in faeces at concentrations 150-fold greater than in the spiked seawater and were defecated for 10 days after removal from *T. gondii*-contaminated water. Oocysts were not detected in snail faeces on days 11–14 following snails’ removal from contaminated water. Error bars denote 1 s.d. from the mean (*n* = 6).

While the ability of EPS-coated surfaces to harvest pelagic particles and retain them in biofilms has been previously reported [37], this process has been overlooked as a mechanism for delivery of terrestrial pathogens to marine benthic communities. We thus conducted laboratory experiments to further investigate the potential for turban snails (*Chlorostoma brunnea, C. montereyi* and *Promartynia pulligo*) to acquire and retain parasites by grazing on EPS biofilms [23]. After a 24 h exposure to *T. gondii* oocysts in tanks containing filtered seawater and kelp, snails were maintained in individual, oocyst-free housing units and their faecal pellets examined over a 14-day period. Oocysts were detected in snail faecal pellets for 10 days post-exposure. No oocysts were detected in the water from the negative control tank or in faecal samples from the negative control snails. Peak oocyst concentrations in faeces were approximately 150-fold greater than the concentrations to which the snails were initially exposed in the water during the 24 h exposure period (figure 4). Microscopically, the defecated oocysts appeared intact, suggesting that the parasite can pass undigested through the snail alimentary canal (figure 3*e*,*f*). Furthermore, snails continued to defecate *T. gondii* oocysts for up to 10 days following removal from the contaminated seawater. Combined, these findings suggest that marine snails can facilitate *T. gondii* transmission to sea otters through parasite bioaccumulation as well as by retention of pathogens, thereby increasing both the dose and duration for risk of exposure to otters. Although gastropods are known to acquire biofilm-associated microorganisms typically found in aquatic habitats (e.g. trematode cysts or mycobacterial cells) [38,39], the link between a benthic-scraping marine invertebrate and transmission of a zoonotic terrestrial pathogen through polymer-mediated mechanisms in the ocean has not been previously reported.

### (c) Implications of polymers for disease transmission in marine ecosystems

Assessing the distribution and fate of zoonotic pathogens in coastal ecosystems is of paramount importance to human public health and wildlife health [1]. Prior studies describe the essential role of polymers including EPS (carbohydrate-rich) and Coomassie stainable (protein-rich) particles in providing micro-island habitats and substrates for aquatic microbes [11,40], yet the role of these polymers in mediating the transmission of pathogens in coastal waters is largely unexplored. As noted, aquatic polymers may facilitate the accumulation of pathogens through two distinct mechanisms: enhanced association with pelagic aggregates and incorporation into biofilms via benthic processes. Moreover, the pelagic process whereby TEPs enhances association of *T. gondii* oocysts with marine aggregates is also relevant to disease transmission in the open ocean: filter-feeding fish and many invertebrates feed on aggregates, and ingestion of zoonotic pathogens associated with these particles may be a critical delivery system for introducing disease agents to higher-trophic-level predators. This process may be especially relevant in explaining the curious pattern of widespread *T. gondii* infections in pelagic marine mammals, including cetaceans [7], which may become exposed through ingestion of aggregate-consuming prey. Indeed, filter-feeding fish such as anchovies and sardines, as well as molluscs (including oysters and mussels), can ingest and retain *T. gondii* oocysts [41–43], thus serving as potential sources of infection to higher-trophic-level predators.

The studies described here used *T. gondii* as a model organism for evaluating terrestrial pathogen transport in coastal ecosystems; however, the polymer-mediated mechanisms identified here are likely to be relevant to disease transmission of numerous terrigenous pathogens that pose health risks to marine hosts or humans. Coastal waters receiving runoff from the land are often polluted [44], yet they provide crucial habitat for wildlife, and are economically and culturally important sources of food and recreation for people. Land-based pathogen pollution can introduce numerous disease agents into coastal waters, including protozoan pathogens such as *Cryptosporidium* and *Giardia* [45], bacterial pathogens like *Salmonella* [46], and viruses including noroviruses—the leading cause of infectious shellfish-born gastroenteritis in humans [47]. Freshwater sources that may transport pathogens to coastal waters are often delivered to piers, coral reefs, kelp beds and tide pools—habitats known to contain rich EPS-producing biota [35,36,48,49]. Thus, coastal waters that receive contaminated runoff may also contain high levels of polymers that could mediate critical disease transmission mechanisms, including control of the spatial distribution and ecological fate of terrestrial pathogens in near-shore habitats. The ramifications of the findings described here extend the role of aquatic polymers in oceanic processes into the domain of epidemiology: invisible colloids, webs, gels and biofilms are likely to be key agents, presently greatly underestimated, that must be considered to successfully address and mitigate pathogen pollution of coastal oceans.

## Supplementary Material

Electronic supplementary material

## Data accessibility

Data have been deposited in Dryad (doi:10.5061/dryad.g0q01) and are in the electronic supplementary material.
