# Supplementary material for: Aquatic polymers can drive pathogen transmission in coastal ecosystems
Source: Proc Biol Sci. 2014 Nov 22;281(1795):20141287. doi: 10.1098/rspb.2014.1287 (PMC4213613; doi:10.1098/rspb.2014.1287)
Supplement: Electronic supplementary material [file rspb20141287supp1.docx]

**Aquatic polymers can drive pathogen transmission in coastal ecosystems**

**Electronic supplementary material (ESM)**

Figure S1. Experiment 1: Mean proportions of *Toxoplasma gondii* oocysts, dragon green (DG), and glacial blue (GB) surrogate microspheres recovered from the aggregate-rich fraction of bottles containing filtered seawater, unfiltered seawater, and seawater spiked with three increasing concentrations of alginic acid (AA) as a source of TEP. Error bars denote SD from the mean (n=5). TEP concentrations are indicated in parentheses as μg Gum Xanthan equivalent L^-1^. Higher numbers of both oocysts and surrogate microspheres were recovered from the aggregate-rich water fraction as TEP concentration in samples increased (negative binomial regression for *T. gondii* oocysts and surrogate particles *P* < 0.001).

Figure S2. Experiment 1: Total aggregate volume (mL) recovered from the aggregate rich fraction of bottles containing filtered seawater, unfiltered seawater, and seawater spiked with three increasing concentrations of alginic acid (AA). Error bars denote SD from the mean (n=5). TEP concentrations are indicated in parentheses as μg Gum Xanthan equivalent L^-1^. For each water type, aggregation control bottles that were placed near the rolling apparatus but not rolled were included; the purpose of these control bottles was to estimate the background levels of aggregates present in seawater with variable TEP concentrations in which aggregate production was not enhanced due to water currents produced by the rolling motion.

Table S1. Mean (SD) of *Toxoplasma gondii* oocysts, dragon green (DG) and glacial blue (GB) surrogate microspheres recovered from the aggregate-rich fraction of bottles containing unfiltered and filtered (0.2 μm) seawater from aggregation experiment 2. Seawater was collected on different months represented by differing phytoplankton assemblages (Table 1). Filtered seawater was used in each experiment as a particle free control to assess oocyst and surrogate distribution in an identical water type that lacks particles that could form aggregates during the rolling process. Filtered control bottles did not contain readily visible aggregates, and oocysts and surrogates were quantified from the same bottom water fraction that corresponded to the aggregate rich fraction of unfiltered samples.

|  | Seawater | | |  | Filtered seawater | | |
| --- | --- | --- | --- | --- | --- | --- | --- |
| Sample collected (2012) | DG | GB | *T. gondii* oocysts |  | DG | GB | *T. gondii* oocysts |
| March | 0.47 (0.02) | 0.32 (0.04) | 0.31 (0.03) |  | 0.12 (0.04) | 0.10 (0.04) | 0.11 (0.01) |
| June | 0.47 (0.05) | 0.40 (0.10) | 0.38 (0.06) |  | 0.11 (0.03) | 0.15 (0.02) | 0.11 (0.03) |
| August | 0.29 (0.06) | 0.27 (0.11) | 0.22 (0.10) |  | 0.07 (0.05) | 0.11 (0.04) | 0.08 (0.02) |

Table S2. Mean aggregate enrichment factors (SD) of *Toxoplasma gondii* oocysts, dragon green (DG), and glacial blue (GB) surrogate microspheres derived from aggregation experiments 1 and 2. In experiment 1, a single sample of seawater (SW) was collected and commercial alginic acid (AA) was added at three increasing TEP concentrations. Experiment 2 utilized seawater containing variable TEP mixtures and concentrations, collected on different months represented by differing phytoplankton assemblages (Table 1). Enrichment factors (EF) depict the numbers of particles per mL of aggregate divided by the number of particles per equivalent volume of surrounding water. Note that EFs decline as a function of TEP in experiment 1, because the overall volume of aggregates (denominator variable) increased with added TEP (Fig. S2). All results are for 24 hr experiments.

|  | Sample  (TEP ug Xeq. L^-1^) | | DG | GB | | *T. gondii* |
| --- | --- | --- | --- | --- | --- | --- |
| Experiment 1 | SW  (332) | | 5.5 X 10^4^ (1.4 X 10^4^) | 1.4 X 10^4^ (6.9 X 10^3^) | | 1.5 X 10^4^ (9.4 X 10^3^) |
|  | SW + AA  (651) | | 5.2 X 10^4^ ( 4.5 X 10^3^) | 1.7 X 10^4^ (1.8 X 10^3^) | | 2.0 X 10^4^ (9.4 X 10^3^) |
|  | SW + AA  (692) | | 1.2 X 10^3^ ( 3.1 X 10^2^) | 4.1 X 10^2^ (1.2 X 10^2^) | | 5.8 X 10^2^ (9.2 X 10^1^) |
|  | SW + AA  (885) | | 2.7 X 10^2^ (3.6 X 10^1^) | 1.3 X 10^2^ (3.5 X 10^1^) | | 1.8 X 10^2^ (2.3 X 10^1^) |
| Experiment 2 | March  (332) | | 5.5 X 10^4^ (6.3 X 10^3^) | 1.4 X 10^4^ (3.1 X 10^3^) | | 1.5 X 10^4^ (4.2 X 10^3^) |
|  | June  (373) | | 3.4 X 10^3^ (1.6 X 10^3^) | 1.9 X 10^3^ (8.7 X 10^2^) | | 2.1 X 10^3^ (8.7 X 10^2^) |
|  | August  (164) | 3.7 X 10^4^ (9.3 X 10^3^) | | 2.8 X 10^4^ (9.2 X 10^3^) | 2.0 X 10^4^ (6.6 X 10^3^) | |
